# The Question of "Gears"

**Published:** 1898-09-10

**Authors:** 


### The Question of "Gears."

In the cycling world few questions are of more interest or more productive of controversy than that of "gears." While the advantages of high-geared machines are fully recognised, and while it is universally admitted that things can be done with them that would be utterly impossible without them, it is maintained by many that all the evil results which cycling is capable of producing fall with special emphasis upon those who use high gear; and this is undoubtedly true. Not, however, we fancy because of the gear, but because those who wish to indulge in high speed, in racing, and in competition—the things which are the real sources of evil—all use high-geared machines.

An interesting paper by Dr. Leonard Williams appears in this month's *Physical Culture*, in which he urges the advantages of high gear. Even in climbing hills he is not inclined to admit its evils. "An indifferent pedaller," he says, "will mount many a hill on a low-gear which he could not possibly climb on a high one, but it is very doubtful whether a really good pedaller ever dismounts to a hill on a high gear which he would have considered it worth his while to ride on a low one . . . . As soon as a person has learned to manage a high-gear he dislikes low gears, and even for hill climbing he does not consider that their advantages outweigh their drawbacks."

Here we seem to see the real bottom of the controversy. It is a question of skill and a question of strength. So far as the mechanics of the machine itself are concerned, a given force applied will produce pretty much the same amount of propulsive effort, whether the gear be high or low. But besides the energy which is used up in moving the machine, a not inconsiderable amount has to be expended in moving the feet at such a speed as to make them bear upon the pedals, and this, when the speed is high, is vastly greater with the low than with the high-geared machines. So short and sudden, in fact, may the movement of the feet have to be to overtake the pedal that proper "ankling" may be quite impossible, and in maintaining sufficient speed, say, to "go without his hands," the rider finds that "it is frequently all that he can do to keep his feet on the pedals at all." With many, indeed, we are told that the power is applied only "by a momentary downward kick at the pedal just after it has passed its highest." Perhaps this is a slight exaggeration, but, at any rate, it shows us what is the true inwardness of this question, namely, that the feet must not be made to move at such a speed as to prevent them from comfortably performing the somewhat complex movements involved in good pedalling. Granting equal skill, then, the gear must bear a proportion to the speed which it is proper to attain, in other words to the strength of the individual.

This brings us back to the therapeutic use of the cycle. There are many people whose powers both as to muscle and heart are very limited, to whom, nevertheless, the use of the cycle is distinctly advantageous so long as they do not attempt more than they can easily do. Such people should choose low-gears, partly as thereby avoiding much temptation, but chiefly because a low-geared machine gives them the rate of pedalling which is appropriate for the highest speed they ought to attempt, and also because, for the very low speeds which the non-athletic person is forced to adopt in going up hill, there is without doubt a distinct economy of energy in a low-gear.

For cases in which the heart is liable to be disturbed, it is again a matter of some importance to bear in mind that the nervous and muscular tension involved in maintaining the balance is an injurious influence, and that, except to those who are highly skilled, this strain is greater, at low speeds, with a high than with a low gear. Nay, we would even say that, so far as the use of the cycle in medicine is concerned, it is a great pity that fashion has set so definitely against the tricycle, a form of machine on which the very lowest of speeds may be indulged in. With a very low-geared tricycle many more or less weakly people, who at present are getting fat in their arm-chairs, could obtain all the benefits, although certainly not all the pleasures, of cycling. The present generation hardly recognises the tricycle, except as a sign of old fogeydom; it forgets that the tricycle was the pioneer, and that much of the advocacy of cycling as a means to health was founded upon experience gained upon the old-fashioned machine. Few medical men did more to popularise the cycle as a means to health than the late Sir Benjamin Richardson. The cycle to him, however, spelled tricycle, and to the day of his death the machine which stood in his hall, and on which he had learned the beneficial effects of cycling, was what the present generation would call "only an old tricycle."

